# A complete dynamical study of time-varying and interconnected networks of pulse-coupled theta neurons

**DOI:** 10.1186/1471-2202-14-S1-P371

**Published:** 2013-07-08

**Authors:** Tanushree B Luke, Ernest Barreto, Paul So

**Affiliations:** 1 School of Physics, Astronomy, and Computational Sciences and The Krasnow Institute for Advanced Study, George Mason University, Fairfax, VA 22030, USA

## 

In 1986, Ermentrout and Kopell[1,2] first introduced a novel mathematical approach to describe the behavior of Type I neurons near their bifurcation point. Starting from these "theta" neurons as basic building blocks, we have developed an exact model[3] that examined the dynamical conditions under which large-scale collective behavior emerges. In addition to being analytically solvable, one key element of our approach was the introduction of heterogeneity within the neuronal population, specifically in terms of individual neuronal excitability. We found that only three steady-state solutions can result from this model: two static equilibrium states and a class of periodic solutions, or limit cycles. Further, we completely classified the dynamical conditions under which transitions between these collective states can occur.

**Figure 1:**
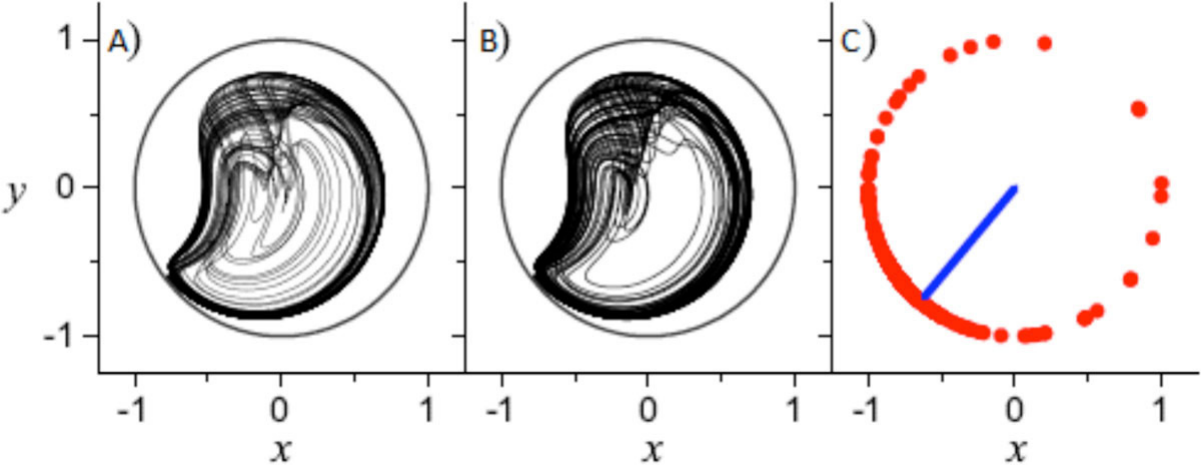
**A) The predicted chaotic attractor obtained from the time-varying reduced equation**. B) The asymptotic trajectory of the macroscopic mean field of the same attractor obtained from a network of 10,000 theta neurons. C) A snapshot showing the instantaneous microscopic phases of 550 randomly sampled neurons, with the instantaneous macroscopic mean field shown in blue.

In our present work, we expand our exact model to examine multi-population networks of theta neurons. In this two population system, each network has heterogeneity not only in its intrinsic excitability, but also in the inter-neuronal coupling between populations (i.e. excitatory vs. inhibitory networks). Here, one population, designated as the "driver", is assumed to be only weakly influenced by the second population, the "response." However, the response is strongly influenced by both its own internal state and the state of the driver, which effectively shifts its median excitability. We find that this shift is constant when the driver is found in an equilibrium state, and we catalog the resulting effects on the state of the response. A periodic steady-state solution from the driver, however, introduces a time-varying excitability in the response. The corresponding behavior of the response mimics that of a parabolic burster network with alternating patterns of recurrent spiking and quiescent periods. In a recent publication[4], we have demonstrated that in such networks, multistability and chaotic behavior (see Figure 1) can result. Here we identify all the macroscopic chaotic states of the response and classify the microscopic states that give rise to these conditions. Further, we also demonstrate the robust nature of these macro-states across any large network of Type I neurons with time varying excitability.
